# Care Delivery Interventions for Individuals with Cancer: A Literature Review and Focus on Gastrointestinal Malignancies

**DOI:** 10.3390/healthcare12010030

**Published:** 2023-12-22

**Authors:** Anh B. Lam, Vanessa Moore, Ryan D. Nipp

**Affiliations:** 1 Department of Medicine, University of Oklahoma Health Sciences Center, Oklahoma City, OK 73104, USA; 2 College of Medicine, University of Oklahoma Health Sciences Center, Oklahoma City, OK 73117, USA; vanessa-moore@ouhsc.edu; 3 Division of Hematology and Oncology, University of Oklahoma Health Sciences Center, Stephenson Cancer Center, Oklahoma City, OK 73104, USA

**Keywords:** gastrointestinal cancer, patient-reported outcomes, hospital-at-home, geriatric oncology, collaborative care, palliative oncology, financial toxicity, supportive care

## Abstract

Background: Gastrointestinal malignancies represent a particularly challenging condition, often requiring a multidisciplinary approach to management in order to meet the unique needs of these individuals and their caregivers. Purpose: In this literature review, we sought to describe care delivery interventions that strive to improve the quality of life and care for patients with a focus on gastrointestinal malignancies. Conclusion: We highlight patient-centered care delivery interventions, including patient-reported outcomes, hospital-at-home interventions, and other models of care for individuals with cancer. By demonstrating the relevance and utility of these different care models for patients with gastrointestinal malignancies, we hope to highlight the importance of developing and testing new interventions to address the unique needs of this population.

## 1. Introduction

The diagnosis and treatment of cancer can pose an abundance of complex challenges for patients, caregivers, and clinicians. Gastrointestinal malignancies represent a particularly challenging condition, often requiring a multidisciplinary approach to management. These challenges may include access to care, persistent symptom concerns, increased healthcare utilization, among others, ultimately leading to a decreased quality of life for patients and their caregivers. Consequently, innovative care delivery interventions are critically needed for patients with gastrointestinal cancer in order to meet the unique needs of these individuals. For example, care models in this field must strive to improve the quality of life and care for patients who often present with a constellation of distinct care needs.

**Figure 1:**
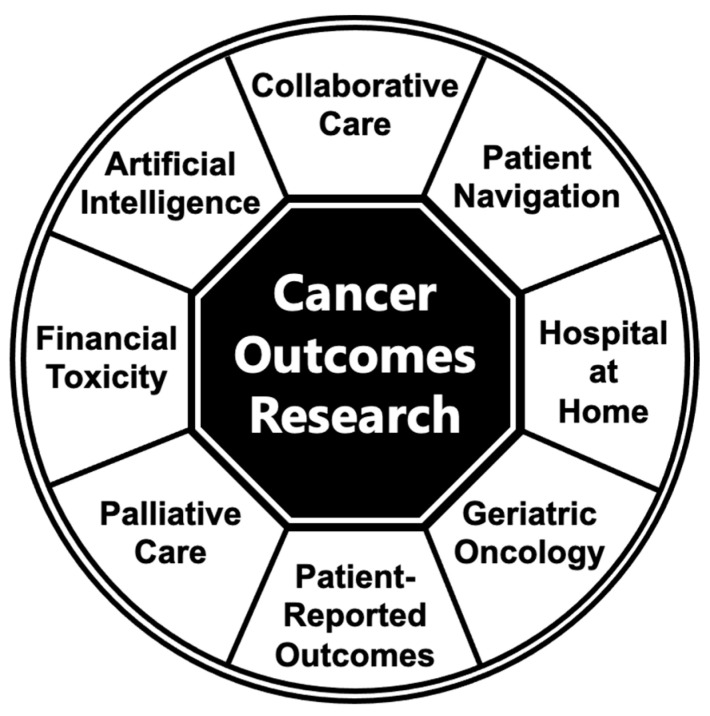
Cancer outcomes research care delivery models.

In this manuscript, we sought to explore the literature regarding models of care delivery interventions focused on issues related to research into cancer outcomes (Figure 1). Specifically, we aimed to describe the existing literature, outline potential problems, and brainstorm future directions among several care delivery interventions. Although not all interventions highlighted in this literature review might be considered innovative and/or specific to gastrointestinal cancer, we hope to motivate ongoing efforts to develop and test novel strategies seeking to enhance care delivery and outcomes for individuals with gastrointestinal cancer by highlighting the potential utility and relevance of these different care models for patients with gastrointestinal malignancies.

## 2. Methods

We searched the literature and used PubMed to find recent studies (within the time period of 2006–2023) focused on several specific topics of interest related to cancer outcomes research and gastrointestinal cancer (Figure 1): gastrointestinal oncology, gastrointestinal cancer, gastrointestinal malignancy, artificial intelligence, patient navigation, patient-reported outcomes, hospital-at-home, geriatric oncology, collaborative care, palliative care, supportive care, financial burden, and financial toxicity. We specifically focused on care delivery interventions, with examples provided in Table 1, along with relevance to patients with gastrointestinal cancer.

## 3. Results

Table 1 provides an overview of example manuscripts describing care delivery interventions, highlights areas of relative innovation among these interventions, and describes the relevance of these interventions to patients with gastrointestinal cancer. Specifically, we focused on care delivery interventions involving artificial intelligence, patient-reported outcomes, hospital-at-home, patient navigation, geriatric oncology, collaborative care, palliative care, and financial toxicity. Within Table 1, we provide details of the number of patients in these example manuscripts, along with the number of participants with gastrointestinal cancers within each study. Notably, this table also describes the relative innovative aspects for each of these selected studies. The remainder of the results below represents our narrative synthesis of the available literature within each of the respective topics.

### 3.1. Artificial Intelligence Interventions

The advances of artificial intelligence (AI) in recent years have led this to become a key part of some of the most popular avenues of expanding technological advancements in healthcare. Hospitals and other healthcare facilities have begun efforts to implement basic technological advancements, which support the future for AI programs, such as the digitalization of medical records, remote appointment scheduling, and automated appointment reminders. These technological advancements have fostered the ability for AI to continue to expand and evolve within healthcare [9]. With the recent opportunities for AI in medicine to expand, particularly in gastroenterology and gastrointestinal oncology, we wanted to explore how AI is currently being used in the field of gastrointestinal oncology and its future potential.

The development of AI models for use in gastrointestinal procedures represents a recent advancement in the field of gastroenterology that continues to undergo new developments. Specifically, the use of computer-aided diagnosis in the detection, staging, and classification of gastrointestinal malignancies has been tested in esophageal, gastric, hepatic, pancreatic, biliary, and colorectal cancers [10,11,12,13,14,15,16]. Interestingly, previous randomized controlled trials using AI computer-aided diagnosis alongside colonoscopies suggested a significantly higher detection rate for colorectal neoplasms than traditional colonoscopy [1,10]. Multiple studies also found that the training of a convolutional neural network to detect Barrett’s esophagus produced the same or higher accuracy rates than its human comparison [17,18,19,20]. With further trials to expand AI developments in endoscopy, the field of gastroenterology’s advances suggest that AI diagnostic models could be used as a supplemental tools alongside physicians’ judgments [21,22,23]. Additionally, these preliminary results suggest that AI could potentially help to overcome observer biases in the diagnoses of gastrointestinal cancers [21].

Challenges in cancer care cover a myriad of complex and nuanced issues, which play roles in the utility of AI in gastrointestinal oncology. For instance, patient–clinician discussions about treatment, side effects, and prognosis frequently require a personalized, tailored approach and a human connection to effectively communicate these difficult topics. Furthermore, the discussion of the risks, benefits, and alternative options related to cancer treatment, and particularly for cancer clinical trials, can be especially nuanced and necessitate a tailored approach [24]. Additionally, with the breadth of information that results from a simple internet search, patients may encounter overwhelming and/or misleading information when attempting to find answers on their own. AI may help patients to overcome this gap, with efforts such as a user-friendly AI search tool developed for patients with cancer that offers tailored searches for clinical trials for which patients may be eligible [24]. Compared to the “clinicaltrials.gov” search engine, this novel AI model created a significantly faster way to identify ongoing trials [24]. This model represents a unique example of how the use of AI may assist with search tools developed by clinicians to enhance patient education and provide information in a more personalized and effective manner.

The development of electronic medical record (EMR) systems has produced a new way of organizing clinical information, yet the EMR may also contribute to the need for more computer support and administrative tasks [25,26,27]. Additionally, time spent on these tasks may impact the time that clinicians spend with their patients. Ambient clinical intelligence (ACI) may help to address these issues, as ACI seeks to understand, adapt, and translate patient–clinician encounters directly into the EMR [26,27]. Although ACI projects are currently underway and have not yet been adopted in routine clinical practice, they hold significant potential to help enhance EMR documentation and increase time spent in patient encounters.

Despite the potential promise of AI, this novel strategy has various limitations and requires continued research to help us to realize its true potential. One concern amongst healthcare professionals and the public alike involves the fear of AI dehumanizing and replacing human interaction(s) in medicine [23,27]. In their development, AI models in medicine are programmed on a statistical and factual basis, which can produce conflict when applying these models to medical diagnoses, an aspect of medicine that is deeply subjective and individualized in terms of disease presentations and treatment planning [28]. Therefore, recent works suggests the need to use AI like an innovative tool rather than replacing the patient–clinician interaction [27,28,29]. However, additional research is needed in order to fully understand the pragmatic use of this tool, as well as the potential benefits for patients, their loved ones, and clinicians alike [9].

### 3.2. Patient-Reported Outcomes Interventions

Assessing patient-centered outcomes, such as symptoms and quality of life (QOL), directly from the patient via patient-reported outcomes (PROs) represents a promising strategy for improving care delivery in the field of gastrointestinal oncology [2,30,31,32]. PROs refer to “measurements of any aspect of a patient’s health status that come directly from the patient [33,34]”. PROs are often used in research, such as clinical trials and novel drug development, to try to help in understanding treatment tolerability and efficacy [34,35,36]. Further, PRO tools can be cancer-agnostic or -specific to the distinct illness type, thereby offering a vast array of options for understanding patients’ perspectives [34]. Recently, research has demonstrated the potential to utilize PROs as a systematic monitoring system to detect problems and address patients’ needs in order to improve their outcomes [37].

A growing body of literature has demonstrated the benefits of using PRO monitoring to enhance QOL, decrease hospitalizations, and potentially improve survival among patients with cancer [2,31,32,38]. For example, a PRO-monitoring intervention that allowed patients to self-report their symptoms and clinicians to proactively address issues that arose resulted in better QOL, fewer emergency department (ED) visits, and decreased hospitalizations [2,38]. In multiple randomized controlled trials, patients assigned to the PRO intervention also experienced longer survival [2,31,32]. PRO-monitoring interventions are also associated with improved symptom control and patient satisfaction [38,39]. Thus, PROs offer a systematic and comprehensive approach to integrating patients’ perspectives into their care, with the expanding literature supporting the potential for PRO-monitoring interventions to deliver a more patient-centered and enhanced-care experience [35,38].

Despite the beneficial associations with PRO monitoring interventions, additional work is needed to fully understand how best to implement PRO monitoring into routine care for patients with gastrointestinal cancers and determine how best to personalize these efforts to patients’ unique needs [34]. Specifically, in order to implement PROs into standard oncology practice, this may require additional buy-in from clinical teams and systems, as well as the need for patients to feel like their efforts to complete the PROs are rewarded by having their clinical team acknowledge and respond to the issues raised [38]. Furthermore, the benefits of PRO-monitoring interventions may differ between certain subgroups of patients, such as older versus younger patients [40]. Moreover, interventions specifically focused on the distinct needs of individuals with gastrointestinal malignancies need to be developed and tested.

### 3.3. Hospital-at-Home Models of Care

Hospital-at-home interventions seek to provide patients with hospital-level care within the comfort of their home [3]. Hospital-at-home care models may benefit both the patient and the healthcare system by improving patients’ QOL while reducing the reliance on hospitals as the only site for hospital-level care [3]. In the field of oncology (and for those with gastrointestinal cancers), hospital-at-home models aim to address patients’ symptoms stemming from the cancer, as well as the side effects of treatment [3]. Consequently, with the complex nature of oncological care, hospital-at-home models represent an innovative strategy with the potential to benefit patients and help to avoid unwanted time away from home.

In gastrointestinal oncology, limited research to date exists that seeks to develop and test hospital-at-home models for patients with cancer. However, several examples exist, including the Huntsman at Home program, the Supportive Oncology Care at Home model at Massachusetts General Hospital, and Penn Medicine’s Cancer Care at Home program [41,42,43]. These models include various different components, such as acute care, symptom monitoring, supportive care, and cancer treatment [44]. Many of these models of care seek to improve QOL, address symptom burden, reduce unplanned hospitalizations, decrease ED visits, and collaterally result in cost reductions [44]. Notably, the Supportive Oncology Care at Home model at Massachusetts General Hospital has specifically focused on patients with gastrointestinal malignancies (namely pancreatic cancer) to date [3]. However, further research is needed to fully understand the potential impact and utility of hospital-at-home interventions in oncology and how best to incorporate these novel models of care into the paradigm of treatment for individuals with gastrointestinal cancer.

Hospital-at-home care for individuals with cancer represents a growing field with immense potential. In combination with PROs, digital health, and telemedicine, these models can augment supportive care strategies for individuals with cancer [3,40,45]. Plausibly, hospital-at-home interventions could help to provide care to patients with cancer in a way that removes barriers to transportation, yet these models may also have challenges and barriers to implementation and access [45]. For example, interventions such as telemedicine have an increasing presence in oncology care, but barriers to this type of care include technological challenges, patient privacy, data security, and resistance to change among patients and clinicians [45]. Collectively, hospital-at-home models of care in gastrointestinal oncology represent a promising strategy, but further research is needed to understand the benefits of these models while also seeking to address potential implementation challenges.

### 3.4. Patient Navigation Models

Individuals with gastrointestinal cancer often face a myriad of challenges, one of which involves navigating the healthcare system. Specifically, patients with gastrointestinal malignancies often have multiple care teams helping to address their cancer, including oncologists, radiation oncologists, surgeons, gastroenterologists, and palliative care clinicians, among various others [46,47,48,49,50]. Thus, the concept of patient navigation has evolved to address the multitude of barriers that can affect access to healthcare and help to support patients throughout their cancer care continuum [50,51].

The existing literature has demonstrated that significant barriers to cancer care can include gaps in health literacy, a lack of transportation, and difficulties with healthcare coverage, among others [50,52]. Consequently, patient navigation models not only seek to help patients to overcome these barriers but also aim to improve clinical outcomes by reducing time to diagnosis, minimizing delays in accessing the continuum of cancer care services, and reducing the number of patients lost to follow-up [49,53,54,55]. Specifically, patient navigation models were developed to address patients’ unique barriers [56,57]. These models offer various methods of patient support and navigation, including psychosocial support, patient empowerment, bridging patients to appropriate resources, and multiple others [56,57,58,59]. Furthermore, existing studies have demonstrated higher rates of colorectal, breast, and prostate cancer screening; increased adherence to follow-up visits; and improved clinical trial accrual as a result of patient navigation interventions [46,47,48,49,53,55,56,57]. Interestingly, not all patient navigation interventions revealed a significant improvement in QOL, likely due to difference in target populations [4]. However, research suggests that patients with cancer and cancer survivors experience improved QOL and increased satisfaction after participating in patient navigation programs, most notably in individuals facing health disparities [48,54,57]. Thus, patient navigation models offer a patient-centered approach within the healthcare system to help patients to overcome challenges in order to improve their outcomes.

The benefits of patient navigation programs have been reinforced by many studies, but the costs and resources required for their implementation may limit access to these programs. Effective strategies to help to demonstrate the cost-effectiveness of patient navigation programs include utilizing community health advisors or patient navigators recruited from within the community [46,54,57]. This not only expands the workforce but also represents an effective strategy for inclusivity by utilizing patient navigators who are bilingual or from within the community [46]. Additionally, patient navigation programs may prove to be profitable by avoiding missed visits and helping to reduce unnecessary health service utilization through patient education and proactive symptom management [56,60]. Notably, further research is needed to develop and test patient navigation interventions to help in understanding the utility and enhancing accessibility among patients with gastrointestinal malignancies.

### 3.5. Geriatric Oncology Interventions

Cancer commonly impacts older individuals (aged ≥65 years), and this population has distinct oncological care needs [61]. More specifically, gastrointestinal malignancies represent some of the most common and problematic cancers in the older adult population [7,62]. The geriatric oncology population presents a unique challenge for the clinicians caring for them, necessitating a comprehensive approach to the management of comorbid conditions, frailty, functional limitations, cognitive changes, polypharmacy, potential limited social support(s), among others [63,64]. In response, geriatric assessment-driven interventions are increasingly being developed and tested in oncology to help to try to meet the needs of older adults with cancer [64,65]. Geriatric assessment often entails formally inquiring about and assessing physical function, falls, frailty, cognition, nutrition, social supports, and polypharmacy [64,66]. Proposed interventions to address geriatric-specific issues frequently involve multi-disciplinary approaches, including physical and occupational therapy, pharmacy, social work, audiology, psychology/psychiatry, dietitian, palliative care, and geriatrics clinicians [64,66,67,68,69]. Thus, older patients often present a unique set of challenges for oncologists, and a comprehensive, multi-faceted approach may be needed to fully meet the needs of the geriatric oncology population, especially considering that older adults represent the largest and fastest-growing group of individuals with cancer [70].

Growing research supports the potential benefits of geriatric assessment-driven interventions in oncology. Specifically, the Comprehensive Geriatric Assessment (CGA) provides an in-depth assessment to help to identify geriatric-specific needs that may not otherwise be identified in routine assessments [71,72]. Incorporating geriatric assessment into oncological care can help to guide treatment decision-making and provide additional supportive care for individual patients [65,71,73]. Research involving geriatric assessment-driven interventions has demonstrated an ability to reduce the occurrence of toxic effects from cancer treatment [5,73,74]. In addition, integrated geriatric assessment interventions may result in improved QOL and reduced healthcare utilization [75]. Interventions that integrate geriatricians into the care of older adults with cancer have shown promise for improving QOL, symptom burden, and even communication amongst patients and their care teams [76]. Further, perioperative geriatric interventions hold great potential to enhance care delivery and outcomes for patients with gastrointestinal cancer undergoing surgery [77]. Therefore, the benefits of geriatric assessment-driven interventions demonstrate encouraging results, with direct applicability for older patients with gastrointestinal malignancies.

Further exploration of the benefits of geriatric oncology interventions are needed to understand how to best implement and optimize efforts to improve outcomes for older individuals with cancer, particularly for those with gastrointestinal cancer. Prior work suggests that novel interventions must sometimes be tailored and adapted for this population [2,31,63]. In addition, ongoing innovations in geriatric oncology are needed to understand additional novel models, such as telehealth and digital strategies; optimal implementation into clinical workflows; and how to best personalize these strategies across different care settings, patient cohorts, and ideal timings of interventions.

### 3.6. Collaborative Care Models

Patients with cancer often need a multifaceted approach to address their myriad of supportive care concerns, including co-existing mental health needs [78,79,80]. For example, in patients with gastrointestinal cancers, the prevalence of depression and anxiety remains high, even after receiving cancer treatment [79,80]. Thus, innovation is necessary, especially in the mental health domain, to address these complex needs of patients with cancer. Collaborative care models represent a potential solution to this challenge. The collaborative care model refers to a systematic approach to proactively treat complex illnesses in the primary care setting, with further promise for the cancer care domain [6,81,82,83,84].

To date, collaborative care models have often entailed a multidisciplinary approach with primary care clinicians, mental health specialists, and care managers collaborating to deliver comprehensive and coordinated care [85,86]. Therefore, collaborative care models have the potential to translate well into cancer care to address the additional mental health needs of this patient population [6,82,83,84,87,88]. At Massachusetts General Hospital, research into a novel collaborative care approach in oncology has sought to improve outcomes by supporting patients with serious mental illnesses and cancer [84]. This collaborative care intervention uses proactive psychiatric involvement to identify people with serious mental illnesses, with patients then being connected to care teams of case managers and psychiatrists who offer additional support with engagement, communication, and psychiatric symptom monitoring throughout the cancer treatment process [84]. Moreover, the University of Washington has implemented the Integrated Psychosocial Oncology Care Program that emphasizes patient-centered and population-based collaborative care [82]. In comparison to standard care, the use of collaborative care models has the potential to reduce rates of depression, both in the short term and the long term, in patients with cancer [6]. Additionally, research into collaborative care approaches suggests these models can help to improve clinical outcomes, promote timely access to care, increase patient satisfaction, and remain cost-effective [87,88].

The efficacy of collaborative care models among patients with cancer has been shown in several studies [6,84,87]. However, gaps still exist in terms of addressing the mental health needs of patients with cancer (particularly those with gastrointestinal malignancies) due to the limited access patients have to these novel care models. While these interventions have evidence suggesting their benefits and potential cost-effectiveness, cancer centers may not have the resources to sustain them [6,87]. These factors include time constraints, financial concerns, limited workforce, and resistance to change [89]. Therefore, addressing these shortcomings in collaborative care models is still necessary to expand its access to patients. Solutions to these barriers will ultimately require gaining support from the healthcare industry to further integrate these models into practice.

### 3.7. Palliative Care Interventions

A key component of caring for individuals with gastrointestinal cancer involves optimizing the delivery of palliative and supportive care [90,91,92]. Palliative care represents a discipline with expertise in symptom management, psychosocial and spiritual care, caregiver support, and end-of-life care, all striving to improve the quality of life and care of patients with serious illnesses [93,94,95,96,97]. Palliative care interventions delivered concurrently with cancer treatment have consistently demonstrated an ability to improve care outcomes in patients with cancer [93,98]. Notably, individuals with gastrointestinal malignancies often have a myriad of palliative and supportive care needs [99]. These include informational needs (understanding of prognosis, advance care planning), physical needs (alleviation of symptoms), emotional needs (coping, arising mental health issues), social needs (caregivers, relationships), and spiritual needs (faith, religion) [93]. Therefore, patients with gastrointestinal cancer experience numerous supportive care concerns, and the appropriate provision of palliative care represents an indubitably important component of care for individuals with cancer.

The existing literature has consistently demonstrated the benefits of the early integration of palliative care for patients with cancer [7,100,101,102,103]. Multiple studies have shown that early palliative care interventions can result in improved QOL, better symptom management, decreased healthcare utilization, and potentially even prolonged survival [100,101,102,104]. Reassuringly, end-of-life discussions were not associated with worsening depressive symptoms in a study of patients treated for non-small cell lung cancer, which should encourage oncologists to execute end-of-life discussions without fear of worsening depressive symptoms [105]. In the same study, oncologists’ compassion was significantly associated with improved QOL and a decrease in patients’ psychological distress [105]. Ongoing work should inform further understanding and navigation of end-of-life discussions in the palliative care setting with patients with gastrointestinal cancer. In patients with gastrointestinal malignancies, palliative care involvement may be associated with less aggressive end-of-life care [92]. Additionally, research has demonstrated benefits of palliative care interventions and caregiver outcomes [104]. Specifically, early palliative care integration can be beneficial for caregivers of patients with gastrointestinal cancers by enhancing their preparedness and readiness to support patients throughout the trajectory of the disease [103,106,107]. Thus, early palliative care involvement allows for patient-directed support and enhances the patient’s and their caregivers’ experience in the setting of an advanced cancer diagnosis.

Future research should aim to study the role, timing, and efficacy of palliative care interventions among patients with gastrointestinal cancers. Notably, prior work suggests that palliative care involvement in real-world settings often occurs late in the disease’s course [91,98,108,109]. Much of the existing research highlights the need for the earlier involvement of palliative care for patients with cancer, but there are limited data to inform the optimal timing and use of palliative care resources in patients with gastrointestinal malignancies. Interestingly, one study of early palliative care demonstrated a significant benefit of the palliative care intervention in patients with lung cancers, but these same benefits were not observed among the patients with gastrointestinal cancer [7]. The underlying mechanism(s) and/or reasons for this finding remain unknown, but this work highlights the need for ongoing research to determine how to best optimize the palliative care efforts across different cancer types, in particular for those with gastrointestinal malignancies.

### 3.8. Financial Toxicity in Oncology

As treatment options for patients with cancer continue to expand, the growing costs of care have become an increasingly significant concern [110,111]. The concept of financial toxicity refers to the financial burden and distress individuals with cancer, their caregivers, and families may face because of cancer care [112,113,114,115]. Contributors to financial toxicity in patients with cancer include prescription drugs, hospitalizations, ED visits, and frequent outpatient visits [116]. Individuals with cancer are also at risk of financial toxicity from loss of income or changes in employment caused by their cancer [112,117]. As a result, major oncology societies, such as the American Society of Clinical Oncology, have published guidelines that recommended costs be considered and discussed with patients [118]. However, limited information exists to guide these cost discussions, and there is scant research surrounding interventions to reduce financial toxicity.

Much of the literature surrounding the topic of financial toxicity has focused on understanding the prevalence and impacts of the financial burden experienced by patients with cancer [116,118,119]. One study found that financial toxicity often co-exists with a multitude of other challenges for patients, such as patient-reported symptoms (pain, fatigue, nausea, depression, anxiety, insomnia), negative illness perceptions, and less confidence in communication [119]. Consequently, researchers have begun seeking to explore ways in which clinicians and patients may tackle this growing problem. A systematic review of clinical guidelines surrounding clinician–patient communication of costs and the financial burden of cancer found consistent themes among the guidelines analyzed [118]. These themes included clinicians’ awareness of price variability between treatment options and insurance coverage, screening for financial stress in high-risk groups, and referral to practical support services to help to alleviate financial burden [118]. Despite the profound impacts that financial toxicity can have in relation to patients’ cancer care, limited research exists to help to guide evidence-based interventions to address this issue.

Future research focused on alleviating financial toxicity in oncology (particularly in patients with gastrointestinal malignancies) should consider prospective, randomized trials of efforts such as cost discussions, financial navigation, and/or proactive financial toxicity monitoring. Prior work suggests that patients have positive attitudes toward cost discussions, and most want cost discussions to take place [120,121,122,123,124], but clinicians may not feel equipped, prepared for, or confident in conducting cost discussions [120,124,125,126]. Strategies entailing the use of financial toxicity screening, such as the use of the Comprehensive Score for Financial Toxicity (COST), to screen patients who may be experiencing financial toxicity merit additional research [127]. One study incorporating financial toxicity screening as a part of PRO monitoring resulted in fewer patients experiencing worsening financial difficulties [8]. Additionally, prior work has piloted a Financial Toxicity Tumor Board as a potential solution for addressing the financial burden experienced by individuals with cancer, and ongoing work should seek to further explore these types of novel ideas [128]. Notably, the idea of financial navigation represents another promising solution for addressing financial toxicity [116,129,130]. A key strategy for financial navigation includes performing comprehensive assessments of patients’ risk factors for financial toxicity, thus allowing financial navigators to offer appropriate support resources or referrals to assist patients with their financial needs [116]. However, ongoing work is needed to help researchers to fully understand the impacts and utility of this type of intervention. Ultimately, future work should aim to address financial toxicity in gastrointestinal oncology at the level of direct patient care, such as providing additional information to coach clinicians in cost discussions and navigating patients throughout their care experience, as well as, on a policy level, calling for change in the affordability of medications and cancer care.

## 4. Discussion

Patients with gastrointestinal malignancies often present with a challenging constellation of symptoms and care needs, frequently meriting a multidisciplinary approach to management. Specifically, individuals with gastrointestinal cancers may experience challenges involving symptom management, access to care, high healthcare use, and complex treatment courses, which may further complicate their ability to maintain a favorable quality of life. Thus, patients with gastrointestinal cancer would benefit from efforts to develop and test care delivery interventions that seek to enhance their quality of life and care.

In the current manuscript, we sought to explore the literature regarding studies of care delivery interventions focused on issues related to research into cancer outcomes (Figure 1). Specifically, we described several care delivery interventions involving artificial intelligence, patient-reported outcomes, hospital-at-home, patient navigation, geriatric oncology, collaborative care, palliative care, and financial toxicity. Importantly, although many care delivery interventions are relevant to patients with various different cancer types, we hoped that the current literature review could help to motivate ongoing efforts to develop and test novel strategies seeking to enhance care delivery and outcomes for individuals with gastrointestinal cancers by describing the potential utility and relevance of these different care models in gastrointestinal oncology.

## 5. Conclusions

The diagnosis of a gastrointestinal malignancy impacts the lives of patients and their caregivers in many ways, and clinicians and care teams are faced with the challenge of meeting these unique needs. We sought to explore the current literature surrounding care delivery interventions for individuals with cancer, with a focus on the relevance and utility of these interventions among gastrointestinal cancers, and our efforts highlight the potential for using various care models to enhance care delivery and outcomes among these patients. By demonstrating the potential impacts of these interventions to improve patients’ quality of life and care delivery, we hope to motivate ongoing efforts to continue to develop and test novel models of care for the gastrointestinal oncology population.

## Figures and Tables

**Table 1:** Example manuscripts describing care delivery interventions.

Care Delivery Intervention	Study	Study Type	Number of Participants	Number of Participants with GI Cancer	Innovation
Artificial Intelligence	Repici et al. [1]	RCT	N = 685	N = 13	Novel use of AI for real-time computer-aided detection (CADe) of polyps, adenomas, and neoplastic lesions during colonoscopy.
Patient-Reported Outcomes	Basch et al. [2]	Cluster RCT	N = 1191	N = 392	Demonstrated the potential for a patient-reported outcome monitoring intervention to enhance physical function, symptom control, and quality of life in patients with advanced cancer.
Hospital-at-Home	Nipp et al. [3]	Pilot	N = 20	N = 20	Revealed the feasibility of delivering an innovative supportive oncology care intervention for patients with pancreatic cancer.
Patient Navigation	Hendren et al. [4]	RCT	N = 319	N = 49	Utilized a randomized controlled trial design to test a novel patient navigation intervention on disease-specific quality of life among patients with newly diagnosed breast and colorectal cancer.
Geriatric Oncology	Mohile et al. [5]	Cluster RCT	N = 718	N = 246	Novel intervention utilizing geriatric assessment tested in a cluster randomized trial and found to reduce treatment toxicity.
Collaborative Care	Ell et al. [6]	RCT	N = 472	N = 55	Demonstrated that collaborative care models are feasible and can help reduce depressive symptoms and improve quality of life in patients with cancer.
Palliative Care	Temel et al. [7]	RCT	N = 350	N = 159	Randomized trial design to test the efficacy of a palliative care intervention among patients with lung and gastrointestinal cancers.
Financial Toxicity	Blinder et al. [8]	Cluster RCT	N = 1191	N = 392	Represents the first financial toxicity screening intervention to show a significant benefit in mitigating financial difficulties in a randomized study.

## Data Availability

Data sharing not applicable.
